# Optimizing the Bioavailability of Subcutaneously Administered Biotherapeutics Through Mechanochemical Drivers

**DOI:** 10.1007/s11095-017-2229-9

**Published:** 2017-07-13

**Authors:** D. S. Collins, L. C. Kourtis, N. R. Thyagarajapuram, R. Sirkar, S. Kapur, M. W. Harrison, D. J. Bryan, G. B. Jones, J. M. Wright

**Affiliations:** 1 Eli Lilly Innovation Center, 450 Kendall Street, Cambridge, Massachusetts 02142 USA; 2 Division of Plastic and Reconstructive Surgery, Lahey Hospital and Medical Center, Burlington, Massachusetts 01805 USA; 3 Clinical & Translational Science Institute, Tufts University Medical Center, 800 Washington St, Boston, Massachusetts 02111 USA

**Keywords:** biodistribution, biologics, drug delivery, lymphatics, subcutaneous

## Abstract

The subcutaneous route offers myriad benefits for the administration of biotherapeutics in both acute and chronic diseases, including convenience, cost effectiveness and the potential for automation through closed-loop systems. Recent advances in parenteral administration devices and the use of additives which enhance drug dispersion have generated substantial additional interest in IV to SQ switching studies. Designing pre-clinical and clinical studies using SQ mediated delivery however requires deep understanding of complex inter-related physiologies and transport pathways governing the interstitial matrix, vascular system and lymphatic channels. This expert review will highlight key structural features which contribute to transport and biodistribution in the subcutaneous space and also assess the impact of drug formulations. Based on the rapidly growing interest in the SQ delivery route, a number of potential areas for future development are highlighted, which are likely to allow continued evolution and innovation in this important area.

## Introduction

The subcutaneous (SQ) route of parenteral drug administration offers numerous benefits, including reduced administration costs, increased patient compliance and preference, and the potential to ultimately integrate drug demand and delivery through closed loop systems, where drug infusion rates are controlled by automated sensors. Challenges in switching from intravenous (IV) to SQ delivery include physically accommodating the large volumes of formulated drugs needed in the SQ space, often lengthy mechanical administration times, and achieving bioequivalence through altered pharmacokinetics (and potentially pharmacodynamics). The latter is a consequence of the SQ environment and variations in rates of blood flow and lymphatic drainage among patients’ physiology and according to injection location. In order to fully realize the potential for SQ drug delivery, thorough understanding of the architecture of the SQ region is required, coupled with comprehension of the myriad parameters influencing drug transport. An ultimate objective would be to establish a set of guidelines pertaining to a given drug, which can be used to model SQ mediated delivery with precise metrics on optimal physical (injection location, depth, and rate) and chemical (drug concentration and formulation) parameters (1). Agnostic of therapeutic area, this review provides a holistic overview of the challenges and opportunities, and outlines potential areas for innovation in this rapidly developing field (2–5).

## Defining the structure of the subcutaneous region

**Fig. 1:**
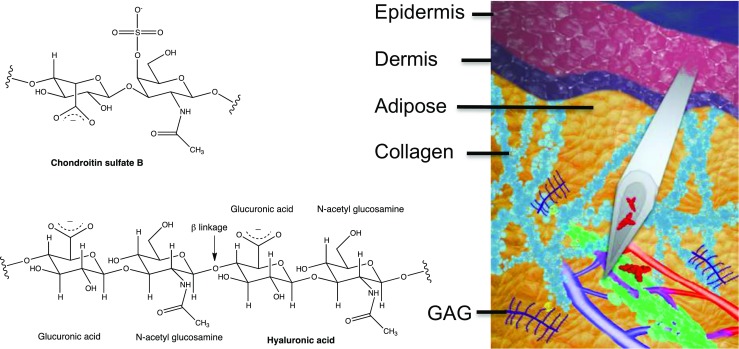
Interstitial matrix components surrounding lymphatic and vascular network (lymphatics = green) for injected drugs (r) and structures of key glycosaminoglycans (l).

The SQ region in humans is a complex, variable domain located between the dermis and muscular layers. Its sequence is comprised of superficial adipose tissue, a fibrous layer of connective tissue (often referred to as the membranous layer), and deep adipose tissue with a boundary to a fascia and the muscle walls (Figure 1). Thickness of the SQ region is dependent on location, personal characteristics, and gender. It increases with body mass index (BMI), decreases with age, and is typically greater in females of comparable BMI These factors are necessarily incorporated into SQ drug delivery regimens. For example, one of the preferred sites for SQ administration of insulin is the abdominal region, which is particularly impacted by patient BMI. This necessitates patient training and rotation of injection sites to maximize biodistribution, and reduce the potential for induration and lipohypertrophy. The use of animal models to mimic SQ drug uptake and distribution has only limited relevance as a major difference to humans lies in the fact that their SQ connective tissue is typically much less rich in fibrous components, presenting a less rigid structure with flexibility to accommodate relatively large volumes of injected solutions with comparative ease (2). Additionally the SQ region in animals presents a pronounced sub-dermal striated muscle known as the *panniculus carnosus*, which can impact studies on injection mechanics, as it is essentially absent in humans (6,7). An outlier to these differences are pigs and mini-pig varieties (8). Having fibrous connective tissue similar to humans, their *panniculus* is not located at the boundary to the dermis, instead separating adipose tissue layers. However, although the subcutis of the porcine model shares many anatomical similarities to human, lymphatic and vascular uptake and subsequent biodistribution can often proceed at markedly different rates (7,9). Accordingly, precise modeling of the human SQ environment is needed (10).

### Chemical components of the interstitial matrix

Injection to the SQ region requires a degree of accuracy to penetrate the epidermal, dermal and pannicular layers while not transitioning to the skeletal muscle below (Figure 1) (3). In humans, 4 mm is an approximate injection depth but ranges from 1–39 mm (average 16 mm) in abdomen, 1–34 mm (average 6 mm) in the arm and 1–32 mm (average 8 mm) in the leg have been reported (4). The subcutis is composed of adipose tissue bound by an extracellular matrix, through which the venous system and lymphatic channels are interspersed, and is enveloped in interstitial fluid derived from plasma. The extracellular matrix is composed of several key macromolecules with unique chemical properties. For example, type I & III collagen is represented by fibrils composed of individual collagen units which are in fact three polypeptide strands (known as tropocollagen) whose triple helices form a quaternary structure stabilized by multiple hydrogen bonds. The high glycine content (every third amino acid) contributes substantively to collagen’s ability to establish key hydrogen bonds and cross links which enhance its mechanical strength. Of significance, the isoelectric point of one of the collagen components (type I) has been calculated to be ~10, thus rendering the fibers net cationic at physiological pH and establishing the potential for attractive interactions with negatively charged matrix components (5). Elastin fibers, formed from multiple 66kD tropoelastin proteins are rich in amino acids which form hydrophobic regions bridged by lysine cross links, which may also foster attractive through-space interactions. Other key macromolecules present in the matrix include proteoglycans and appended glycosoaminoglycans (GAG’s). Examples of GAG’s include heparin, an oligosaccharide (average MW ~12-14kD) composed of sulfated α 1,4 linked units of iduronic acid and glucosamine, which bears a net negative charge as a consequence of the ionized sulfate moieties. Chondroitin is another key GAG, exemplified by chondroitin sulfate B (sometimes referred to as dermatan sulfate) a variable mass oligosaccharide composed of GalNAc or GlcA linked via β1,4 or 1,3 linkages, which is known to engage in multiple binding interactions with cytokines, matrix components and growth factors, and plays a key role in wound healing and tissue damage (11). Per building block, chondroitin bears two ionizable sites per repeated unit, comprising both carboxylate and sulfate moieties and rendering the oligomer highly anionic in physiologic pH (carboxylate pKa ~3–5, sulfate pKa ~1.5–2) (12). Hyaluronic acid (often referred to a hyaluronan) is another negatively charged component with a pKa of 2.9 in physiologic pH (13), and is of renewed significance, as its enzymatic degradation [typical MW = 6–8 x 10^6^] via injected hyaluronidases is a method used to enhance uptake and trafficking of SQ injected drugs [vide infra] (14). Interestingly and significantly, though hyaluronan represents approximately 1% of the concentration of collagen in skin, its fluid exclusion volume potential is *ten* times that of collagen (15). Solutions of hyaluronic acid can be highly viscous, and as molecular weight increases it adopts a spherical conformation with a hydrodynamic volume of ~600 nm for a 10^6^ MW oligomer (14). Hyaluronic acid is also hygroscopic and contributes to the viscoelasticity of skin. Its properties are related to molecular weight, the native polymer having defined structural properties whereas oligomers can vary and are responsible for numerous biologic events including proliferation of endothelial cells and cell migration (16). Interactions between GAG’s is also possible, and it has been suggested that the viscosity of hyaluronic acid is enhanced by chondroitin sulfate (17).

### Subcutaneous adipose tissue

Surrounding the complex labyrinth of fibers, proteoglycans and GAG’s are variable deposits of adipose [fat] tissue, and the entire region is bathed in interstitial fluid. Adipose layers can be characterized as either deep, membranous or superficial layers and thickness is dependent on patient BMI and location (6). Of significance for drug delivery, an immunostaining study showed the presence of critical lymphatic vessels is highest in the dermis and fascia regions but low in superficial and deep adipose layers *independent* of patient BMI (10). Models have been developed to interrogate injection physiology into adipose tissue. On injection, hydraulic fracturing of the tissue results in micro-cracks, which can increase permeability to injected fluids. X-ray imaging methods have been used to visualize the injected plume, which initially adopts a conical formation then begins to disperse (18). Adipose tissue is classified into white adipose and less abundant but mitochondria-rich brown adipose tissue (BAT), which is becoming of renewed importance in diabetes research (6,19). White fat cells are 90–99% triglyceride, 2–3% protein and 5–30% water (20), and in obese patients, remodeling of the extracellular matrix is common to accommodate growth of adipocytes which may have the potential to contribute to patient variability in SQ drug uptake as evidenced by studies on insulin (21,22).

### The vascular and lymphatic systems

Interspersed within the SQ tissue is the inter-related network of arteries, veins and lymphatic channels. Transport of ISM solutes into the vascular system is influenced by oncotic pressure exerted by dissolved proteins in the surrounding interstitial fluid and the uptake of lower molecular weight injected drugs via endothelial cells is now well understood (23). In the case of larger biomolecules however, absorption through the lymphatic network is the predominant pathway and involves a complex interplay of mechanical and chemical processes (6,10,24,25). Lymphatic capillaries (with high surface area) are abundant in dermal layers, and transdermal delivery has indeed been exploited (26), but in the case of large molecular weight biomolecules and antibodies, the volumes required to mirror doses delivered by IV methods are sufficiently large to render transdermal delivery impractical. Lymphatic vessels however are abundant in the SQ layers, albeit present a lower surface area (relative to capillaries) for drug uptake, necessitating additional considerations for large volume capture and transport. The lymphatic system originates as a network of capillaries which transport fluid from dermal layers and the SQ interstitium (6,10,24). The capillary networks (or initial lymphatics as they are sometimes referred to) drain into lymphatic collecting vessels, several of which feed into afferent trunks which in turn connect to lymph nodes (Figure 3). Nodes are often interconnected in regional groups. From the nodes, fluid is transported via efferent trunks towards the thoracic duct, entering venous circulation at the intersect of the left subclavian and jugular veins (24).

### Injection site remodeling events

An additional consideration for SQ drug administration in chronic diseases is the potential for tissue induration and scarring at the injection site (27). Similarly, for the SQ administration of large formulated drug volumes in short time periods, pressure build up at the injection site can cause complications including leakage and tissue scarring as a consequence of hydraulic forces (28). Future developments of ‘closed loop’ SQ drug delivery systems using implanted devices will also have to address an additional injection site event known as the ‘foreign body reaction’. In this situation a fibrous network develops as a consequence of injection site inflammatory response, limiting drug perfusion (29).

## Transport of biomolecules in the subcutaneous region

On injection to the SQ region low molecular weight drugs and proteins (<16kD) can be absorbed through capillaries and then enter systemic circulation (2). Since their size precludes entry however, higher molecular weight proteins and antibodies must traffic through the lymphatic system where they enter general circulation at the interface of the thoracic lymph duct with the subclavian vein (3). As lymphatic flow rates can vary between 0.2% and 2% to that of blood, understanding lymphatic uptake is of key importance (2).

### Drug uptake through the lymphatic system

**Fig. 2:**
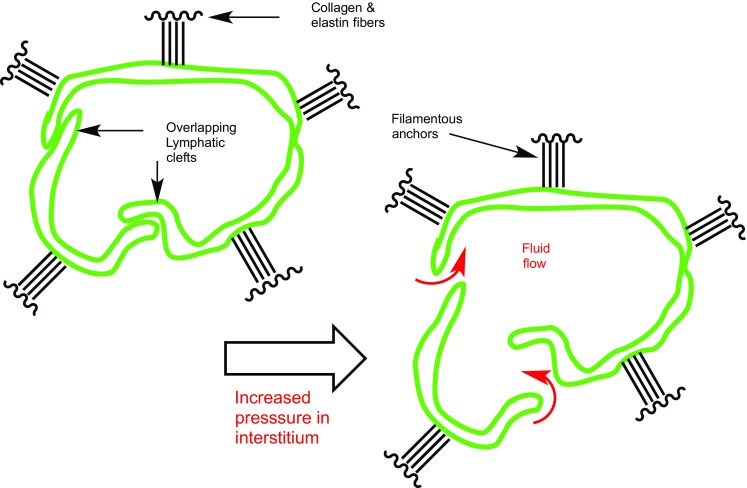
Endothelial lumen and clefts involved in fluid transport to lymphatic system.

Movement within the interstitial matrix involves a combination of diffusion and convection, and transport models between components of the interstitium have been developed based on the Starling equation and the Brinkman and Darcy equations (30,31). Interstitial fluid traffics through the lymphatic system at a rate of 0.2-1 μm/s, the movement being dependent on pressure gradients between the lymphatic system and interstitium. A commonly accepted model suggests cleft like junctions open between lymphatic endothelial cells in the capillaries to allow passage of macromolecules into the lymph system (Figure 2), with expansion from 10 nm to over 1000 nm based on chemical and physical gradients, influenced by attached collagen and elastin fibers (2,32,33). This picture may underestimate the complexity of the process however (3,5,34,35), and the possibility of active transport through numerous endothelial cell surface proteins (e.g. cadherins, catenins) in both the vascular and lymphatic systems are an under explored strategy, as are charge-based gating pathways (36). Another interesting option could be to actively target and exploit the FcRn neonatal receptor, (which is known to play a significant role in the SQ uptake of monoclonal antibodies) through chimeric affinity constructs (37).

### Mechanical stimulation of the lymphatic system

**Fig. 3:**
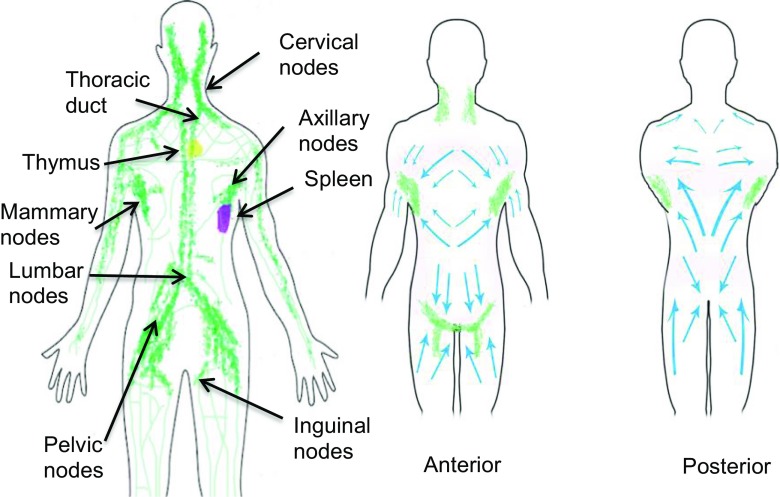
Lymphatic ducts and nodes (left) and lymphatic drainage points (right).

Interstitial fluid dynamics and lymphatic flow are known to be both temperature and exercise dependent which has been attributed to filtration forces acting across capillary walls (38). Tissue compression [e.g. when walking] increases interstitial flow whereas stretching impedes this in a process that may be governed by fibroblasts (39). The impact of these mechanical phenomena on SQ drug dispersion and uptake has not been extensively studied though models have been developed (40). In an effort to promote lymphatic flow [and thus drug uptake] it is also possible to employ other mechanical methods. Clinical studies have been conducted on the impact of manual lymph drainage (MLD) in fibromyalgia patients (41). The methods, which include use of soft tissue massage regimens, targeted physical exercise [e.g. yoga], use of graduated compression bandages and pneumatic devices (e.g. the Lympha Press system) have shown a dramatic increase in lymph flow, reducing edema and other clinical markers (Figure 3) (42). Studies have also shown synergistic impact of ultrasound with manual drainage to increase lymph flow (43), which may afford additional benefits. The connective tissues are not fully hydrated at physiological state, and compression/stretching cycles results in substantial flux as GAG’s attract water based on their net negative charges.

### Phoretic stimulation of lymphatic uptake

Given the impact of hydration of GAG’s on their function and properties, sonophoresis, which is known to impact intra and inter-molecular hydrogen bonding networks, is likely to have a pronounced impact on drug dynamics. Indeed, in addition to disrupting hydrogen bonding networks of oligosaccharides (44), sonophoresis has been shown to reduce solution viscosity (45), and promote localized thermally induced massaging effects which may also improve flow (2,46). Elsewhere, the impact of sonophoresis on SQ and adipose tissue is routinely exploited during abdominoplasty surgery, where it is used to aid mechanical dissolution of adipose tissue via lipolysis (47). Given that (degraded) lipids are actively up taken by the lymphatic system it may be insightful to study post-sonophoretic lymphatic activity in control subjects not undergoing subsequent adipose aspiration surgery. Additional possibilities include the use of radio-frequency induced thermal effects (48), which are routinely used in cosmetic dermatology procedures to effect skin-tightening and re-sculpting, assumed to occur via FM based interaction with dermal collagen (49). Iontophoresis is another method that can enhance the transport of charged molecules through the skin and the subcutaneous space, demonstrated most notably with anti-inflammatory agents (50,51). This can be achieved by applying a continuous low-voltage current of appropriate polarity. Highly charged drugs can be transported by means of electrophoresis, whereas uncharged compounds transport can be enhanced by the electroosmotic flow of water assisted by the dissolved mobile cations (e.g. Na + or K+) movement (51). However, there are limitations to the size of the molecules that can be transported. One of the benefits of iontophoresis, is that the rate of drug transport can be controlled at will by adjusting the electrical current which is correlated to the drug flux almost linearly.

### Modulating interstitial pressure

Interstitial pressure increases rapidly when solutions of drugs are injected SQ, and a variety of methods can be used to quantitate (52). Elevated pressures in the interstitium and at the injection site can increase pain nociception and thus means to dissipate large injected boluses are of interest. Administration of the enzyme hyaluronidase (e.g. rHuPH20) reduces this pressure substantially and with rapid onset, by enzymatic degradation of hyaluronan, thereby reducing viscosity in the interstitium (52). The co-administration of this enzyme in clinical studies has been successfully demonstrated through IV to SQ non-inferiority trials with various biologics, a function of its ability to act as a volume expander (53). Though initial concerns regarding the potential for immunogenic response had been raised (54), clinical application in immunoglobulin therapy (55,56), and oncology (57) has been demonstrated and additional applications can be anticipated (58). Use of hyaluronidase to enhance perfusion and distribution of injected solutions dates several decades (59). With initial studies [using non recombinant sources of enzyme], repeat administration resulted in hypersensitivity towards the agent (59). In addition to application in drug delivery however, it has also been employed under off-label use in cosmetic surgery to modulate the physical effects of injected hyaluronic acid based ‘fillers’ (60,61). This is potentially significant, as the enzymatic reaction is considered reversible, tempting speculation that the degraded components may in theory subsequently *reform* high molecular weight oligomers and polymers of hyaluronic acid (60). Additionally, it has been suggested that the presence of very high molecular weight hyaluronic acids confers cancer chemoprotective properties in some species (62). The impact of altering the mass distributions of hyaluronic acids through enzymatic processes and homeostatic processes (baseline turnover of HA is around 1/3 per day in humans) (63) may thus become an area of importance for long term clinical studies.

### Movement through the ISM

The movement of biomolecules through the interstitial matrix is impacted by electrostatic interactions, and the pI of the protein though important, is not always a predictor for transport dynamics. This is because in the ISM, the hyaluronic acid [and other GAG’s] functions as a polyelectrolyte, and even close to the protein pI, local charges on the protein can foster associations with the ISM components (64,65). In terms of the interstitial volume, it is known that the β1 integrin receptor causes contraction of collagen fibers resulting in compaction of collagen gels. The process is governed by fibroblasts, and during inflammation, inhibition of the integrins allows tissue expansion and concomitant fluid influx, resulting in swelling and edema (66). Deliberate administration of an anti β-integrin IgG likewise promotes influx and edema suggesting this may be a druggable target (66). Interstitial flow is a complex process with multiple components, and as a result algebraic approaches to modeling [akin to principal component analysis] need to be continually developed and refined to help establish criteria that can be used to predict drug transport at the level of the individual patient (67).

### Injection site considerations

The actual site of SQ administration is of relevance in addition to the depth of injection, and may have marked, patient-specific impact in terms of drug bioavailability in comparison to alternate delivery modes (68). Rotation protocols employed to reduce induration and edema (an example being studies on Bortezomib) (69) also need to be influenced by optimal access to the lymphatic system itself as shown on studies of human growth hormone (70), erythropoietin (71) and insulin (72). Systems level mapping of the human lymphatic system has been conducted providing insight to lymph collectors and connecting nodes (35,73). For example, in the anterior abdominal wall, the lymphatic channels are in the midline watershed and periumbilical regions in the subdermal plane (6,10,24). However, in the lower abdomen the lymph collectors lie deeper in the SQ tissue as they travel inferiorly, penetrating Scarpa’s fascia approximately 2–3 cm superior to the inguinal ligament before emptying into the inguinal lymph nodes (Figure 3) (6,10,24,74). Accordingly, based on collector location, bioavailability would be predicted to be higher in the mid-abdominal periumbilical region compared to when injected into the lower abdomen (24,72). This can be expected to be a fertile area for future investigation, as studies to date have been inconclusive (2), or focused solely on clinical outcomes (53,68–72). Imaging at the tissue level of lymphatic duct density will also be useful in order to prioritize injection sites. Lymphoscintigraphy, commonly used to assess lymphedema and lymph associated metastases by assessing gross transport to the lymphatic nodes, has obvious potential in mapping the lymphatic microenvironment and the impact of long term SQ drug administration in chronic conditions (73,75). Another useful tool will be to establish anatomical maps of soft tissue massage points which can be used to mechanically stimulate lymphatic flow to aid in drug uptake (Figure 3). Developing appropriate methods to image the distribution of drug & vehicle boluses from the SQ tissue into lymph are also important. Aside from scintigraphy, basic echography has recently proven useful and is commonly accessible in healthcare settings (76).

### Age related anatomical changes

Given that transport in the SQ environment involves mechanical forces, it is expected that the fidelity and efficiency of the processes will naturally alter with patient age. For example, reduction of both SQ adipose tissue (77) and hyaluronic acid levels (78) have been associated with aging. Hyaluronic acid levels in the epidermis begin to diminish in the 60–80’s which impacts the function of fibroblasts, eventually reducing tissue elasticity as collagen level production falls (78). Though collagen has a half life of approximately 15 years, hyaluronan is under 24 h (15), its age-associated reduction, MW distribution and composition linked to catabolism and degradation via the hyaluronan synthase family of enzymes (79). Injected native hyaluronic acid [exogenous] is degraded rapidly, thus to increase and maintain levels (79), regulation of dermal fibroblast function is instead necessary or else cross-linked hyaluronic acid introduced (which when used in cosmetic indications typically has persistence of 9–12 months). Another concern relates to drug catabolism at the SQ delivery site, which can diminish bioavailability (2). Adipose redistribution takes place in old age and has many potential implications, including metabolic dysfunction and inflammatory processes which might impact the fidelity of SQ delivered drugs (80), and also hyaluronan related repair at the injection site (78). Accordingly, the administration of SQ drugs in chronic diseases afflicting the elderly [Alzheimer’s disease being a prime candidate] needs to be informed by these anatomic and physiologic alterations in terms of drug potency, formulation and injection site rotation (81).

## The impact of formulations on bioavailability

Charge, molecular weight, formulants, pH, temperature, viscosity and tonicity all play a role in the PK and PD of injected biopharmaceuticals (2,3,5). Given the impact of hydration and charge on ECM and interstitium components and their transport properties, it is imperative that formulation of SQ delivered drugs is considered appropriately. In addition to the active drug substances, the nature of buffers used and their relative tonicity (23) impacts biodistribution and lymphatic uptake of biopharmaceuticals (82). Numerous studies have examined the role of buffer and substrate charge, pI and tonicity and their impact on uptake and pK, with variable results (83). In one example, the use of a charged buffer, O-phosphoserine, enhanced lymphatic uptake of an antibody (23). Anecdotally, the hyaluronic acid GAG bears negative charge, thus positively charged species may associate (thereby reducing lympatic uptake) whereas negatively charged species could traffic more rapidly to the lymph based on repulsive Coulombic forces. The role of additives have been explored, including use of the protein albumin to enhance drug uptake, by acting as a volume expander in the ISM in a similar manner to hyaluronidases (84). Active albumin mediated *uptake* into the lymphatic system can also be exploited, by derivitizing the drug in question with an albumin binding glyceride motif, effecting what has been dubbed ‘albumin hitchiking’ (35). Potentially relevant, association of serum albumin with immunoglobulins (through non specific binding events) is known to impact certain immunoassays, and should be examined in formulated drugs using relevant methods (85).

### Predictive modeling

**Fig. 4:**
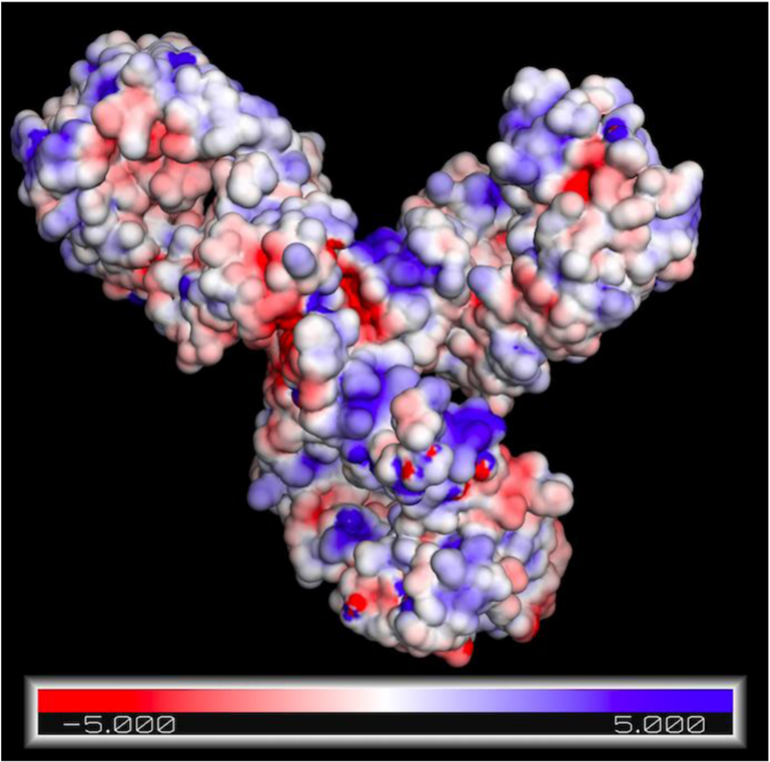
Electrostatic potential map of Pembrolizumab (sequence 5DK3, PDB) rendered in PyMOL (92).

A primary objective will be to predict the interaction of candidate biopharmaceuticals with various ISM components and incorporate this methodology into drug screening assays and selection. This will allow modeling of how they can be expected to transform in the subcutaneous milieu (1). To achieve this requires deep understanding of fundamental properties of the drug and how these impact its bioavailability. For example, a useful tool for drug development would be to correlate zeta potential and isoelectric point with viscosity of the drug (86). This might be used for the re-engineering of antibodies with more desirous properties (87), including solubility (88). It is also important to assess the nature of any charged variants of proteins which might impact affinity and transport in the ISM using appropriate techniques (89). In this regard, the assessment of charge remains a critical yet ill-defined parameter. Electrophoretic methods have been described to quantitate the Debye-Huckel-Henry charge, and studies of the colloidal properties of proteins reveal that proximity energies, which are electrostatic in nature, play a dominant role (90). This is important, as the typically highly concentrated formulations used can lead to the formation of aggregates, gels and emulsions (90). Interactions between [charged] protein drugs and cellular macromolecules is also important. It has been revealed that despite charge variances and regional permutations, anions selectively and preferentially accumulate on the surface of proteins in salt solutions (91). Accurate modeling of the electrostatic surface of proteins might then be used to assess potential interactions with matrix components (Figure 4). Though charge based interactions between proteins can be attractive or repulsive [important for trafficking to the lymphatic from the interstitial matrix], so-called ‘excluded volume’ interactions are always repulsive in nature. Of available methods, electrophoresis can be used to calculate protein valence using the equation, where μ represents electrophoretic mobility, z_eff_ corresponds to the effective valence, f signifies translational friction coefficient, and Q_p_ fundamental proton charge (93).

Collectively, we anticipate that these design considerations will pave the way for what will eventually become known as ***qu***antitativ***e s***ubcutaneous ***t***argeting (QUEST) strategies.

$$ \upmu ={\mathrm{z}}_{\mathrm{eff}}/\mathrm{f}\;{\mathrm{Q}}_{\mathrm{p}} $$

### Considerations for monoclonal antibodies

In the case of monoclonal antibodies, interactions of formulated drugs with buffers and excipients are vastly different in serum. Methods to study this include analytical ultracentrifugation [AUC] using fluorescence detection (94). At high antibody concentrations, both the nature and magnitude of intermolecular interactions is a key parameter impacting its viscosity, and charge distribution plays a pivotal role (95). A primary concern with antibodies is the potential for aggregate formation, as this can have immunogenic consequence (96). At low concentration, changes in conformational stability and weak protein-protein interactions can induce aggregation, and various techniques including dynamic light scattering can be employed to monitor these events (97). It has also been demonstrated that SQ delivery of highly concentrated formulations of mAb’s can be achieved using crystalline suspensions (98), and more recent investigations have been reported on mAb based gel beads (99). An increasingly common strategy is the derivitization of proteins as poly ethylene glycol conjugates (PEGylation). This can have the impact of increasing the Stokes radius of a given protein, and methods have been developed to assess the impact of this modification on properties, including hydrodynamic behavior (100). Though PEGylation of certain proteins has been shown to increase SQ mediate lymphatic uptake, in the case of antibody derivatives the impact is less pronounced, but has been shown to enhance plasma clearance in rodent models (101). It has also been postulated that PEGylation of certain SQ delivered nano-encapsulated drugs results in accelerated blood clearance through intravenous pathways, suggesting potential for synergistic enhancement of drug delivery (102).

Ultimately it may be possible to model potential interactions *in silico*, prior to investing in costly preclinical programs. Aggregation of proteins at high salt concentration, a known problem in drug development, can be measured *in vitro* but may eventually be predictable based on charge density mapping (103), as might viscosity (104). Likewise, protein-protein interactions can be modeled using light scattering techniques and can give insight to potential limitations of a drug candidate (105). Finally, molecular dynamics simulations, previously unimaginable studies on antibodies, are now proving insightful, and with the advent of ever increasing computational methods and processing power, can be expected to play a role making meaningful assessments of drug candidates (106). A major concern for the injection of high molecular weight antibodies in concentrated form is precipitation at the injection site, which can occur through steric exclusion processes involving GAG’s and formulants (107). One means to assess potential for this is to inject constituted drugs *ex vivo* into tissue, then section the tissue using MALDI-MS, scanning for aggregates (108). Also to be considered is the potential to impact the local environment with co-administered adjuvants. For example lipolysis in fat cells can occur via stimulation of β-adrenoceptors, which might be used to alter lipid levels during drug administration e.g. using isoprenaline (109). The degradation of adipose tissue (similarly enhanced by sonophoretic lipolysis during abdominoplasty) may enhance bulk distribution of injected drugs at the site of lipolysis as the degraded products [glycerol] may have a stimulatory impact on lymphatic flow. Likewise, addition of anti β-integrin IgG to induce edema/influx and promote uptake of the drug may become a viable strategy, allied to injection site modeling. Conversely, as trafficking to the lymphatic system from the ISM relies on aqueous transport, hydrophobic components of the formulated drug are likely to accumulate in the matrix, may impact dispersion of the injected bolus and contribute to aggregation (99).

## Strategy and Outlook

The SQ drug administration route is becoming of increased significance in the delivery of biopharmaceuticals. Several compounds administered by the route are now in clinical use (53,110,111), and long term studies are beginning to validate the merit of hyaluronidase based co-formulations (55). Realizing the full potential of subcutaneous delivery methods is likely to have a pronounced impact on patient care and disease management (112) and it is incumbent on the pharmaceutical industry to devise strategies which fully exploit this potential (113). When incorporated into closed-loop automated delivery systems, this may open the possibility of achieving homeostasis by dosing (through pulsatile processes) from a reservoir to the interstitium. The lymphatic system, the main route for uptake of biologic drugs, flows at a much lower rate than via capillary uptake however. Algorithms used to predict, monitor, and analyze kinematics of drug flow will become necessary and may be informed by other systems – e.g. the Riemann method for modeling merged traffic flow (114), biologic filtration bed processes (115) and porous drain engineering principles (116), which rely on the Darcy and Brinkman equations (30). Ultimately systems may emerge that allow us to stress test candidate biomolecules using simulated subcutaneous environments and closed loop drug delivery platforms (1,117).

Based on findings in this rapidly unfolding field, it seems likely that certain areas will prove fertile in the search for long term solutions, as outlined in Table I.

**Table I:** Potential Areas for Future Research and Innovation in SQ Drug Delivery

1. accurate determination of net charges on candidate proteins and formulants
2. the impact of mechanical forces (massage, stretching) on lymphatic flow and drug uptake
3. the impact of hydration, and disruption of hydrogen bonding (ultrasound) in drug dispersion and uptake
4. the potential for electrical stimulation of drug uptake via pulsed and linear processes
5. thermal processes that improve drug uptake and patient nociception (e.g. RF)
6. processes which enhance dissipation of injected plumes to lymphatic capillaries
7. the impact of adjuvants to enhance drug uptake including
-integrin receptor inhibitors to induce edema / drug influx
-lipolysis inducers for drug dispersion and uptake
-endothelial targeting agents which promote lymphatic cleft opening and trafficking
-analgesics which increase patient pain thresholds for the introduction of large injected volumes (118)
-co-administration of protease inhibitors to enhance bioavailability (119)
8.Exploring the utility of mAb-FcRn targeting chimeras to enhance lymphatic uptake

Equally important will be parallel long term studies on the impact of rHuPH20 and volume expanders on interstitial and lymphatic integrity. It has been suggested that enzymatic degradation of the interstitium leads to collapse of the lymphatic vessels (32). Designing *in vivo* lymphatic imaging experiments, and high resolution analysis of basement membranes e.g. using SEM techniques may reveal the impact of the enzyme in appropriate detail. There may be additional avenues to enhance drug uptake and permeation, including the use of encapsulated vectors which possess charges (or can be induced on demand) to exploit Coulombic forces in the interstitium (120). Another strategy may be via induction of accelerated blood clearance (ABC) pathways through derivatization of the drug substance itself (104). We look forward to the incorporation of these and related ***qu***antitativ***e s***ubcutaneous ***t***argeting (QUEST) strategies in mainstream drug development. Given the evident commercial opportunities in this space (121), innovations are likely to proceed at a rapid pace and will form the cornerstone of a new era in patient engaged drug delivery.

## Abbreviations

ABC: Accelerated blood clearance
AUC: Analytical ultracentrifugation
BAT: Brown adipose tissue
BMI: Body mass index
ECM: Extracellular matrix
FM: Frequency modulated
GAG: Glycosaminoglycan
HA: Hyaluronic acid
ISM: Interstitial matrix
IV: Intra venous
MALDI: Matrix assisted laser desorption ionization
MLD: Manual lymph drainage
MS: Mass spectrometry
MW: Molecular weight
PD: Pharmacodynamics
PDB: Protein database
PEG: [Poly]ethylene glycol
PK: Pharmacokinetic
QUEST: Quantitative Subcutaneous Targeting
RF: Radio frequency
SEM: Scanning electron microscopy
SQ: Subcutaneous
